# Machine learning model with *post-hoc* SHAP interpretation based on perioperative clinical and imaging parameters for prediction of 1-year radiographic recurrence after hallux valgus surgery

**DOI:** 10.3389/fsurg.2026.1987508

**Published:** 2026-10-21

**Authors:** Xuefeng Luo, Xieraili Alimu, Aikebaierjiang Aisaiti

**Affiliations:** 1 Department of Trauma Orthopedics, The First Affiliated Hospital of Xinjiang Medical University, Urumqi, Xinjiang, China; 2 Department of Orthopedics, Xinjiang Uygur Hospital, Urumqi, Xinjiang, China

**Keywords:** hallux valgus, machine learning, model interpretability, radiographic recurrence, SHAP, temporal-validation

## Abstract

**Objective:**

Radiographic recurrence after hallux valgus surgery is an important issue affecting long-term imaging outcomes. Previous studies have suggested that preoperative deformity, postoperative residual HVA/IMA, and sesamoid reduction are associated with recurrence, but nonlinear relationships may exist among multiple parameters. The aim of this study was to establish and temporally validate a machine learning prediction model based on conventional perioperative clinical and imaging parameters and to provide *post-hoc* model interpretation using SHAP.

**Methods:**

597 patients undergoing hallux valgus surgery in the Department of Trauma, First Affiliated Hospital of Xinjiang Medical University from 2021 to 2025 were retrospectively enrolled. Radiographic recurrence was defined as HVA >20° on the fixed 12-month postoperative weight-bearing anteroposterior radiograph. Patients operated from 2021 to 2023 formed the development cohort (*n*=389), and patients operated from 2024 to 2025 formed the independent temporal-validation cohort (*n*=208). Eighteen candidate predictors were screened in the development cohort using collinearity assessment, LASSO, RFE, and permutation importance. A unified eight-feature set was then locked and used for comparison of LR, RF, XGBoost, LightGBM, and SVM using 5-fold outer/3-fold inner nested cross-validation conditional on the fixed feature set. Models were evaluated using ROC-AUC, PR-AUC, Brier score, calibration, and DCA, with *post-hoc* SHAP-based interpretation of the final SVM.

**Results:**

A total of 109 patients met the 1-year radiographic recurrence criterion (18.3%). Eight variables were retained: age, preoperative HVA, preoperative IMA, preoperative DMAA, 6-week postoperative HVA, 6-week postoperative IMA, 6-week postoperative DMAA, and 6-week postoperative sesamoid grade. The development-cohort ROC-AUCs of LR and SVM were 0.908 and 0.906, respectively, with corresponding PR-AUCs of 0.766 and 0.757. SVM was retained as the final model on the basis of its lower Brier score and overall performance profile, without claiming statistical superiority over LR. In the temporal-validation cohort, SVM ROC-AUC was 0.944 (95% CI 0.908–0.973), PR-AUC 0.805, Brier score 0.071, sensitivity 0.892, and specificity 0.842. SHAP showed the largest model contributions for 6-week IMA, HVA, and sesamoid grade.

**Conclusion:**

The SVM model with *post-hoc* SHAP-based interpretation showed promising performance for prediction of fixed 1-year radiographic recurrence using routine clinical and perioperative imaging parameters. Residual IMA, HVA, and sesamoid grade at 6 weeks were leading predictive signals within the model rather than causal determinants of recurrence. The model may support early risk stratification and enhanced follow-up, but multicenter external validation, reproducibility assessment, and prospective impact studies remain necessary before clinical implementation.

## 1 Introduction

Hallux valgus (HV) is a common forefoot deformity characterized by varus deviation of the first metatarsal, lateral deviation of the hallux, rotation/pronation of the first metatarsal, and relative displacement of the sesamoid complex (1). Epidemiological studies have shown that HV has a high prevalence in the general population and is more common in women and older adults (2). Surgical correction remains an important treatment for patients with pain, shoe difficulties, or significant dysfunction who fail to respond to conservative treatment; Chevron, Scarf, proximal metatarsal osteotomy, and Lapidus procedures may be selected according to deformity severity and first-ray stability (3, 4).

Although modern surgical techniques can substantially improve HVA, IMA, and symptoms, postoperative radiographic recurrence remains a major concern affecting long-term outcomes. Systematic reviews indicate that recurrence rates vary according to the definition used, surgical method, and follow-up duration. A meta-analysis of 23 studies including 2,914 patients reported an overall recurrence rate of approximately 24.9%, and HVA thresholds around 20° are among the most commonly used radiographic definitions of recurrence (5). Therefore, identifying patients at higher risk of future radiographic recurrence early after surgery may help guide follow-up intensity. Previous studies have reported associations of larger preoperative HVA and IMA, postoperative residual HVA/IMA, insufficient sesamoid reduction, metatarsus adductus, and joint incongruence with recurrence (6, 7). These factors are interrelated, and conventional univariate analyses or fixed thresholds may not adequately reflect their combined predictive information. Machine learning can integrate multidimensional variables and capture potential nonlinear relationships. In 2024, Zhao et al. (8) developed a hallux valgus recurrence model using radiographic data from 198 feet across two centers and reported an external-validation AUC of 0.88, supporting further investigation of machine-learning approaches in this setting.

Clinical prediction models require attention not only to discrimination but also to calibration, clinical net benefit, and transparent interpretation (9, 10). Based on real clinical data from the Department of Trauma Orthopaedics of the First Affiliated Hospital of Xinjiang Medical University, this study used temporal validation, nested cross-validation for candidate-model comparison, multi-method feature screening, and *post-hoc* SHAP interpretation to establish a combined clinical and imaging prediction model. We hypothesized that a multiparameter model would identify fixed 1-year radiographic recurrence risk and that residual deformity measures at 6 weeks postoperatively would contribute substantially to prediction.

## 2 Methods

This retrospective study was approved by the Institutional Ethics Committee of the First Affiliated Hospital of Xinjiang Medical University(Approval No. K202411-28);in view of the retrospective design of this study and the complete anonymization of patient data prior to analysis,the committees waived the requirement to obtain informed consent from each patient. All study procedures were performed in strict compliance with the Declaration of Helsinki and relevant institutional guidelines.

### 2.1 Data sources and inclusion criteria

This single-center retrospective cohort study enrolled consecutive adult patients who underwent primary hallux valgus surgery in our hospital from January 2021 to December 2025. Throughout this report, n denotes patients, and the analytical dataset contained one patient-level record with one recorded operated side per included record. Inclusion criteria were: (1) age ≥18 years; (2) primary hallux valgus surgery, regardless of surgical method; (3) complete preoperative, early postoperative (6-week), and fixed 12-month postoperative weight-bearing foot anteroposterior radiographs; and (4) complete clinical and perioperative imaging data required for the candidate predictors. Exclusion criteria were: (1) revision surgery or previous ipsilateral foot surgery; (2) hallux valgus secondary to inflammatory arthropathy, neuromuscular disease, or trauma; (3) missing key imaging parameters before or at 6 weeks after surgery; and (4) follow-up shorter than 12 months or loss to follow-up. The retained source record begins with the finalized 597-patient analytical cohort and does not contain the upstream screening log; consequently, the total number initially screened and criterion-specific exclusion counts cannot be reconstructed from the materials available for revision. The same source record does not preserve whether any patient underwent bilateral procedures during the overall study interval or how an index side would have been selected. These reporting gaps are stated explicitly rather than inferred retrospectively.

All cases were divided into two non-overlapping cohorts according to operation time: patients operated between January 2021 and December 2023 were included in the development cohort (*n* = 389), and patients operated between January 2024 and December 2025 were included in the temporal-validation cohort (*n* = 208). The temporal-validation cohort was evaluated only after the analysis plan, final feature set, candidate-model comparison, hyperparameters, and classification threshold had been fixed using development-cohort information. No temporal-validation performance metric was used to replace the model, adjust parameters, or rescreen variables. The primary outcome was HVA >20° on the fixed 12-month postoperative weight-bearing anteroposterior radiograph; the 12-month measure was used only for outcome determination and was not a candidate predictor (Figure 1).

**Figure 1:**
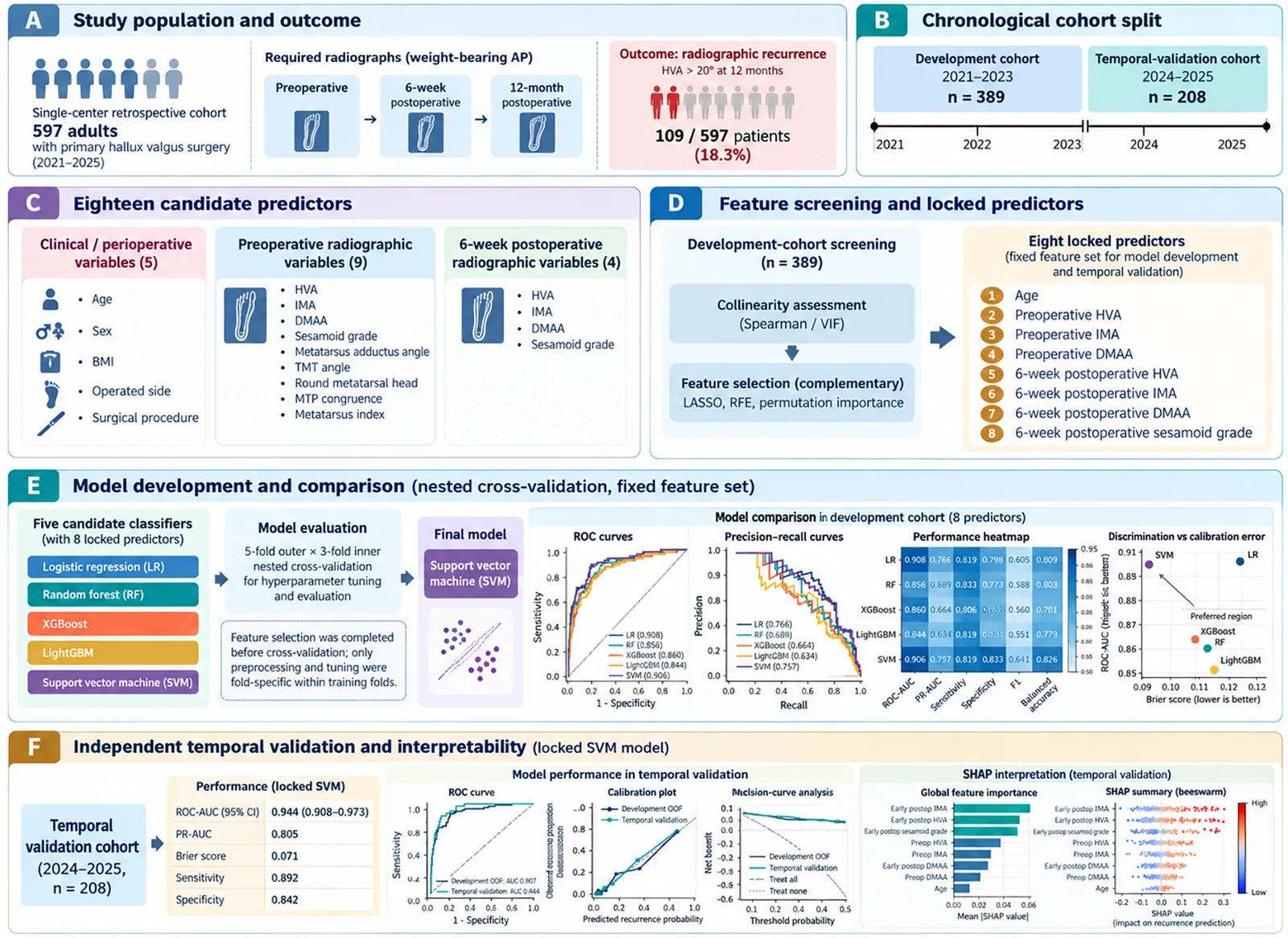
Study design and machine-learning workflow for predicting radiographic recurrence after hallux valgus surgery. **(A)** Study population and outcome. **(B)** Chronological cohort split. **(C)** Eighteen candidate predictors. **(D)** Feature screening and selection of eight predictors. **(E)** Model development and comparison using nested cross-validation. **(F)** Independent temporal validation and SHAP interpretability analysis. Twelve-month radiographs were used solely for outcome assessment, not as predictors.

### 2.2 Radiographic assessment

Weight-bearing anteroposterior (AP) foot radiographs were obtained preoperatively, at 6 weeks postoperatively, and at the fixed 12-month postoperative assessment according to the center's standardized imaging protocol. Preoperative and 6-week images were used to extract candidate predictors, whereas the 12-month image was used only to determine the primary outcome and was not entered into the predictive models. HVA was defined as the angle between the longitudinal axes of the first metatarsal and first proximal phalanx; IMA as the angle between the longitudinal axes of the first and second metatarsals; and the distal metatarsal articular angle (DMAA) as the angle between the distal articular-surface orientation of the first metatarsal and a line perpendicular to its longitudinal axis (11). Sesamoid position was graded from 1 to 7 according to the tibial sesamoid position relative to the longitudinal axis of the first metatarsal on the AP radiograph using the Hardy-Clapham grading system (12). Metatarsus adductus angle, first tarsometatarsal (TMT) angle, first metatarsal head lateral-edge morphology (round/non-round), first metatarsophalangeal joint congruence, and metatarsal index were assessed on preoperative radiographs. HVA, IMA, DMAA, and sesamoid grade were measured at 6 weeks as candidate predictors of early postoperative residual deformity (Figure 2). The retained image-reading record does not preserve reader identities or experience, blinding status, duplicated independent readings, or a dedicated reliability sample. Study-specific intraobserver/interobserver ICCs for continuous measurements and weighted kappa for ordinal grading therefore cannot be reconstructed retrospectively. Published hallux valgus reliability studies and general reliability guidance support explicit reporting of ICC model/type/definition and kappa-based agreement for ordinal ratings (13–15); the absence of a study-specific reliability analysis is therefore acknowledged as an important limitation.

**Figure 2:**
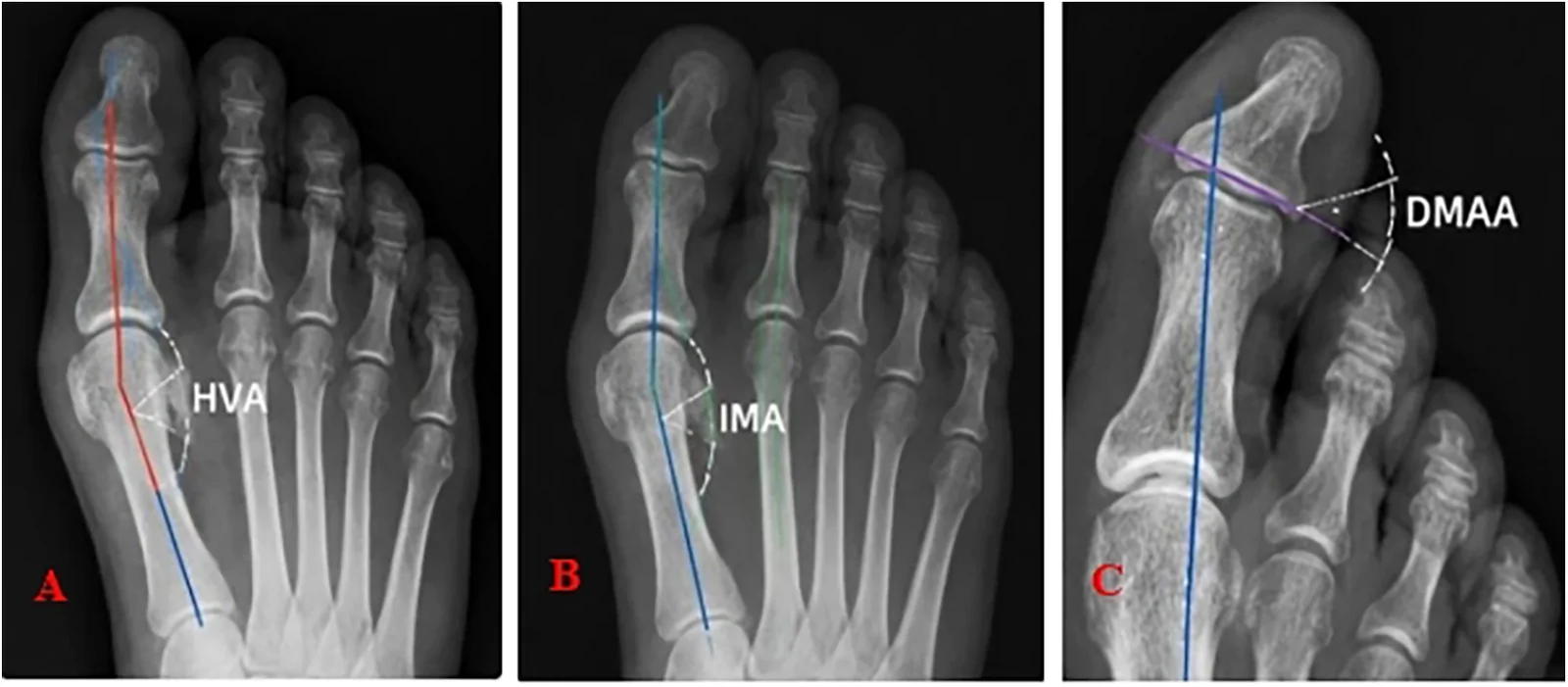
Radiographic measurements of hallux valgus parameters: **(A)** hallux valgus angle (HVA); **(B)** first-second intermetatarsal angle (IMA); **(C)** distal metatarsal articular angle (DMAA).

### 2.3 Outcome definition

The primary outcome was radiographic recurrence at a fixed 12-month postoperative time point, defined as HVA >20° on the weight-bearing AP radiograph obtained at that visit. The >20° threshold was selected because it is one of the most commonly used radiographic definitions of hallux valgus recurrence in the published literature and was used in multiple studies summarized in a prior systematic review and meta-analysis (5). The 12-month HVA and variables derived from outcome information were not used as predictors. Additional available follow-up beyond the 12-month assessment was used only descriptively and did not redefine the binary primary outcome; patients were classified according to the fixed 12-month assessment rather than later radiographs. The analytic archive designates this as the 12-month visit, although the exact scheduling tolerance around the nominal visit was not preserved in the retained record. Thus, the study should be interpreted as a fixed-time-point binary prediction analysis rather than a survival or time-to-event analysis.

### 2.4 Candidate predictor variables

Predefined eighteen candidate variables were determined in advance. Clinical variables include age, gender, BMI, surgical side, and surgical procedure; preoperative imaging variables include HVA, IMA, DMAA, sesamoid grade, metatarsus adductus angle, TMT angle, rounded metatarsal head, first metatarsophalangeal joint matching, and metatarsus index; postoperative six-week variables include HVA, IMA, DMAA, and sesamoid grade. Continuous variables were retained in their continuous form. The correction amounts of HVA/IMA/DMAA and the improvement amount of the sesamoid were derived mathematically from preoperative and postoperative parameters,and were not included simultaneously with their constituent variables to avoid redundant information;there is a deterministic relationship between the lengths of the first and second metatarsals and the metatarsus index, thus the metatarsus index was retained in the primary model.

### 2.5 Data preprocessing and multicollinearity

Eighteen candidate predictive variables had no missing values in the finalized 597-patient analytical dataset. Completeness was assessed before temporal cohort splitting, and no imputation was performed. Continuous variables were standardized for LR and SVM using parameters estimated from the relevant training data. Sex, surgical side, metatarsal-head morphology, and first metatarsophalangeal joint congruence were represented as binary 0/1 variables; surgical procedure was one-hot encoded, and individual category/dummy levels were not counted as separate candidate predictors. During nested cross-validation, preprocessing parameters were fitted on the relevant training portion and then applied to the corresponding validation fold. Spearman correlation coefficients and VIF were used to evaluate collinearity; the maximum absolute correlation coefficient was approximately 0.169 and the maximum VIF approximately 1.43, indicating no severe collinearity.

### 2.6 Feature selection strategy

Feature screening was restricted to the development cohort and did not use information from the temporal-validation cohort. LASSO (16), recursive feature elimination (RFE), and permutation importance were treated as complementary screening tools rather than as a strict intersection or voting rule. The available method-specific outputs are shown in Figure 3: LASSO cross-validation and coefficient trajectories (Figures 3A,B), RFE performance across retained feature counts (Figure 3C), and permutation importance ranking (Figure 3D). Permutation importance ranked 6-week postoperative IMA, 6-week postoperative HVA, 6-week postoperative sesamoid grade, preoperative HVA, and preoperative IMA as the five leading variables in the archived output. RFE showed only small performance changes around an eight-feature solution, supporting a parsimonious model size of eight. The unified feature set was therefore derived by considering LASSO coefficient stability, the RFE performance plateau, permutation-importance ranking, clinical availability at the 6-week prediction time point, nonredundancy, and the number of recurrence events. The final set comprised age, preoperative HVA, preoperative IMA, preoperative DMAA, 6-week HVA, 6-week IMA, 6-week DMAA, and 6-week sesamoid grade. The retained analysis archive does not contain a feature-by-feature membership matrix for the original LASSO and RFE runs, so such membership is not reconstructed *post hoc*. Importantly, the eight-feature set was selected once using the full development cohort and then held fixed for candidate-model comparison. A fully leakage-protected workflow would repeat data-dependent feature selection inside each outer training fold; therefore, the reported nested-cross-validation performance should be interpreted as conditional on the locked development-derived feature set and may retain some optimism.

**Figure 3:**
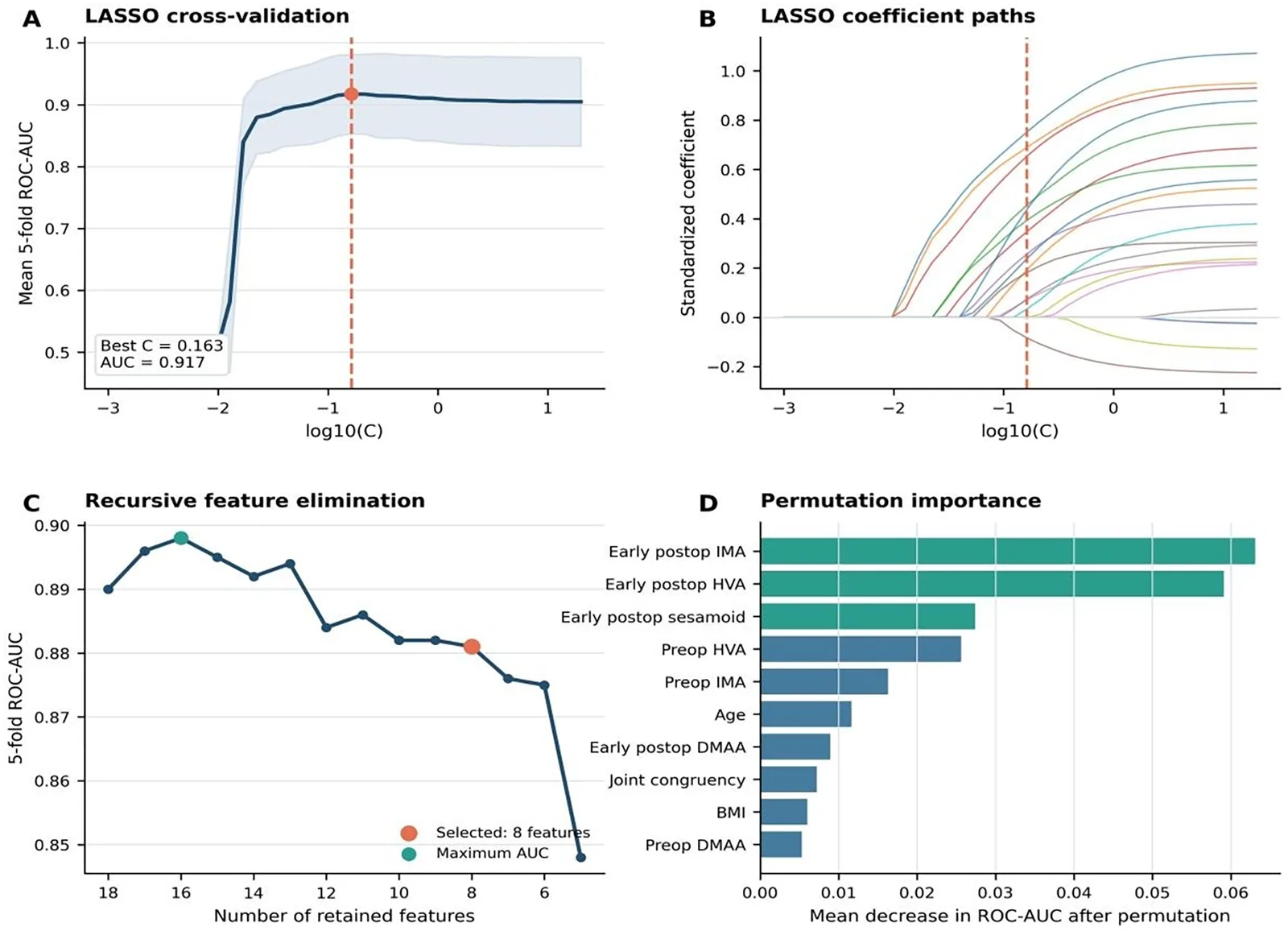
Feature selection and dimensionality reduction results: **(A)** LASSO cross-validation curve; **(B)** LASSO coefficient path; **(C)** recursive feature elimination performance across feature counts; **(D)** permutation importance. These outputs were used as complementary development-cohort screening information and were not combined by a strict intersection rule.

### 2.7 Model establishment and nested cross-validation

With the final eight-feature set held fixed, binary classification models were constructed using LR, RF, XGBoost, LightGBM, and SVM (17). The actual archived analysis sequence was: development-cohort feature screening → lock the eight-feature set → 5-fold outer cross-validation; within each outer training split, training-derived preprocessing and 3-fold inner hyperparameter tuning were performed → outer-fold evaluation → lock the final model and operating threshold → one-time application to the temporal-validation cohort. Thus, nested cross-validation was used for model tuning and performance estimation conditional on the fixed feature set rather than for a fully nested feature-selection pipeline. The retained final SVM used an RBF kernel with C = 0.2 and gamma=0.03 together with class weighting for the approximately 18% recurrence prevalence. The complete candidate hyperparameter grids for all five algorithms, random-seed log, repeated-cross-validation schedule beyond the stated 5-by-3 design, and per-outer-fold performance table were not preserved in the retained analysis archive; these settings are therefore not reconstructed retrospectively.

### 2.8 Performance evaluation, calibration, and clinical utility

ROC-AUC and PR-AUC were emphasized jointly as discrimination measures, with PR-AUC particularly informative given the approximately 18% recurrence prevalence. Sensitivity, specificity, PPV, NPV, F1 score, and balanced accuracy were also reported. Probability-prediction quality was evaluated using the Brier score, with calibration assessed by calibration curves, calibration intercepts, and calibration slopes. Decision Curve Analysis (DCA) compared the model with treat-all and treat-none strategies over threshold probabilities of 0.05–0.50 (18, 19). This range was interpreted as a hypothetical decision range for considering enhanced follow-up rather than an indication for automatic revision surgery. The classification threshold of 0.172 shown in the retained analysis output was locked before application to the temporal-validation cohort. A high-risk classification was intended to prompt enhanced clinical follow-up and repeat weight-bearing radiographic assessment. The retained analysis output does not preserve the exact criterion used to derive the 0.172 operating threshold or whether a separate *post-hoc* probability-calibration transformation was applied; therefore, these settings are not assigned retrospectively. After final model locking, the model was applied once to the temporal-validation cohort, and 95% confidence intervals for temporal-validation performance metrics were estimated by bootstrapping. Because the study did not test a management strategy triggered by model output, DCA findings are interpreted as potential decision utility rather than demonstrated clinical benefit.

### 2.9 Explainability analysis

SHAP was used for *post-hoc* interpretation of the final SVM (20). The development cohort was used as the interpretation background, and the independent temporal-validation cohort was used to generate the reported interpretation results. Global importance and direction of contribution were visualized using mean |SHAP|, beeswarm, and dependence plots, while local contribution plots were used for representative high-risk and low-risk cases. For nonlinear models such as an RBF-kernel SVM, the SHAP framework supports model-agnostic kernel-based explanation (20). The retained analysis archive, however, does not preserve the exact explainer object, background-sample count, target output scale (probability versus decision function), or other computational settings; these implementation details are therefore not reconstructed *post hoc* and are acknowledged as a reproducibility limitation. A locally weighted linear surrogate analysis following the LIME framework was used only as a supplementary model-agnostic explanation and was not interpreted etiologically.

### 2.10 Statistical analysis

Continuous variables are described using medians (interquartile ranges), and group comparisons are performed using the Mann–Whitney U test;categorical variables are presented as counts (percentages),and comparisons are conducted using the *χ*^2^ test or Fisher's exact test. A two-sided *P* value of less than 0.05 is considered statistically significant. Variable selection in predictive modeling is not strictly based on a single-factor *P* value of less than 0.05.The study was written in accordance with the TRIPOD + AI guidelines (9),and also drew upon the standardized reporting framework for interpretable machine learning in clinical research (21).

## 3 Results

### 3.1 Patient characteristics and recurrence status

A total of 597 patients were enrolled, all of whom had 6-week postoperative imaging and the fixed 12-month radiograph required for primary-outcome assessment. According to the predefined criterion of HVA >20° at 12 months, 109 patients experienced radiographic recurrence, giving a 1-year recurrence rate of 18.3%. In the development cohort of 389 patients, 72 had radiographic recurrence (18.5%); in the temporal-validation cohort of 208 patients, 37 had radiographic recurrence (17.8%). Additional available follow-up ranged from 12 to 54 months, with a median of approximately 23 months, but later follow-up did not alter the fixed 12-month primary outcome. The recurrence group was older and had more pronounced preoperative and 6-week postoperative deformity. Preoperative HVA was 38.6° (33.9–42.7°) in the recurrence group and 32.6° (28.7–37.8°) in the non-recurrence group; 6-week postoperative HVA was 13.0° (10.6–16.4°) and 9.2° (6.6–12.4°), respectively; and 6-week postoperative IMA was 8.6° (7.1–9.9°) and 6.5° (5.3–7.8°), respectively. The retained manuscript output does not provide the variable-by-variable development-versus-temporal cohort summaries required to calculate standardized differences, so the earlier broad statement of cohort equivalence has been removed rather than reconstructed. With only 37 recurrence events in the temporal-validation cohort distributed across several surgical procedures, procedure-specific or severity-stratified discrimination/calibration estimates would be imprecise and were not interpreted as definitive subgroup results (Table 1).

**Table 1:** Clinical and perioperative imaging characteristics in the recurrence group versus the non-recurrence group.

Variable	Overall (*n* = 597)	Non-recurrence (*n* = 488)	Recurrence (*n* = 109)	*P* value
Age (years)	50.0 (43.0–58.0)	50.0 (43.0–58.0)	53.0 (47.0–59.0)	0.009
BMI (kg/m^2^)	23.9 (21.9–25.9)	23.9 (21.9–25.9)	24.6 (22.4–26.0)	0.063
Preoperative HVA (°)	33.9 (29.3–39.2)	32.6 (28.7–37.8)	38.6 (33.9–42.7)	<0.001
Preoperative IMA (°)	13.7 (11.6–15.9)	13.4 (11.3–15.6)	15.4 (13.3–17.2)	<0.001
Preoperative DMAA (°)	13.0 (10.0–16.0)	12.5 (9.7–15.8)	14.5 (11.4–17.8)	<0.001
Preoperative sesamoid grade	4.0 (3.0–5.0)	4.0 (3.0–5.0)	5.0 (4.0–6.0)	<0.001
Preoperative metatarsus adductus angle (°)	18.6 (15.3–21.9)	18.2 (14.9–21.3)	20.5 (17.7–23.9)	<0.001
Preoperative TMT angle (°)	3.3 (1.5–4.9)	3.1 (1.4–4.7)	3.9 (2.5–5.6)	<0.001
Metatarsal index (mm)	−2.4 (−6.7–2.1)	−2.5 (−6.9–1.8)	−2.3 (−5.6–3.2)	0.162
6-week postoperative HVA (°)	10.0 (7.1–13.1)	9.2 (6.6–12.4)	13.0 (10.6–16.4)	<0.001
6-week postoperative IMA (°)	6.8 (5.6–8.3)	6.5 (5.3–7.8)	8.6 (7.1–9.9)	<0.001
6-week postoperative DMAA (°)	7.8 (5.8–10.0)	7.5 (5.3–9.5)	9.6 (7.7–11.8)	<0.001
6-week postoperative sesamoid grade	2.0 (1.0–3.0)	2.0 (1.0–3.0)	3.0 (2.0–4.0)	<0.001
Sex				0.710
Female	508 (85.1%)	417 (85.5%)	91 (83.5%)	
Male	89 (14.9%)	71 (14.5%)	18 (16.5%)	
Surgical side				0.204
Left	304 (50.9%)	242 (49.6%)	62 (56.9%)	
Right	293 (49.1%)	246 (50.4%)	47 (43.1%)	
Surgical procedure				0.508
Chevron osteotomy	183 (30.7%)	154 (31.6%)	29 (26.6%)	
Lapidus procedure	59 (9.9%)	45 (9.2%)	14 (12.8%)	
Other osteotomy	35 (5.9%)	27 (5.5%)	8 (7.3%)	
Proximal metatarsal osteotomy	73 (12.2%)	57 (11.7%)	16 (14.7%)	
Scarf osteotomy	247 (41.4%)	205 (42.0%)	42 (38.5%)	
Round metatarsal head				0.048
0	396 (66.3%)	333 (68.2%)	63 (57.8%)	
1	201 (33.7%)	155 (31.8%)	46 (42.2%)	
First metatarsophalangeal joint congruence				0.036
Congruent	325 (54.4%)	276 (56.6%)	49 (45.0%)	
Incongruent	272 (45.6%)	212 (43.4%)	60 (55.0%)	

Continuous variables are presented as median (interquartile range, IQR), and categorical variables as *n* (%). Intergroup comparisons used the Mann–Whitney U test for continuous variables and Pearson chi-square or Fisher exact tests for categorical variables. *P* values were two-sided, with <0.05 considered statistically significant. BMI, body mass index; HVA, hallux valgus angle; IMA, intermetatarsal angle; DMAA, distal metatarsal articular angle; TMT, tarsometatarsal joint; IQR, interquartile range.

### 3.2 Feature selection results

A total of 18 candidate clinical and radiographic predictors available by the 6-week prediction time point were prespecified. These comprised age, sex, BMI, surgical side, surgical procedure, preoperative HVA, IMA, DMAA, sesamoid grade, metatarsus adductus angle, TMT angle, round metatarsal head, first metatarsophalangeal joint congruence, metatarsal index, and 6-week postoperative HVA, IMA, DMAA, and sesamoid grade. RFE showed only small changes in cross-validated AUC around an eight-feature solution, and the final unified set comprised age, preoperative HVA, preoperative IMA, preoperative DMAA, 6-week HVA, 6-week IMA, 6-week DMAA, and 6-week sesamoid grade (Table 2, Figure 3). In the archived permutation-importance output, the five leading variables were 6-week IMA, 6-week HVA, 6-week sesamoid grade, preoperative HVA, and preoperative IMA. Table 2 reports one row per predictor family so that one-hot category levels are not miscounted as separate candidate predictors. Method-specific screening outputs are presented in Figure 3 and were interpreted as complementary rather than as a strict intersection rule; a feature-by-feature LASSO/RFE membership matrix was not available in the retained analysis archive.

**Table 2:** Feature selection results for candidate variables.

Candidate predictor	Representation	Time point	Final status	Comment
Age (years)	Continuous	Clinical	Retained	Final 8
Sex	Binary 0/1	Clinical	Not retained	One predictor family
BMI (kg/m^2^)	Continuous	Clinical	Not retained	
Surgical side	Binary 0/1	Perioperative	Not retained	One predictor family
Surgical procedure	One-hot encoded	Perioperative	Not retained	Dummy levels not counted separately
Preoperative HVA (°)	Continuous	Preoperative	Retained	Final 8
Preoperative IMA (°)	Continuous	Preoperative	Retained	Final 8
Preoperative DMAA (°)	Continuous	Preoperative	Retained	Final 8
Preoperative sesamoid grade	Ordinal 1–7	Preoperative	Not retained	
Preoperative metatarsus adductus angle (°)	Continuous	Preoperative	Not retained	
Preoperative TMT angle (°)	Continuous	Preoperative	Not retained	
Round metatarsal head	Binary 0/1	Preoperative	Not retained	
First MTP joint congruence	Binary 0/1	Preoperative	Not retained	
Metatarsal index (mm)	Continuous	Preoperative	Not retained	
6-week postoperative HVA (°)	Continuous	6 weeks	Retained	Final 8
6-week postoperative IMA (°)	Continuous	6 weeks	Retained	Final 8
6-week postoperative DMAA (°)	Continuous	6 weeks	Retained	Final 8
6-week postoperative sesamoid grade	Ordinal 1–7	6 weeks	Retained	Final 8

All 18 predictor families entered development-cohort screening. The final status column refers to the unified eight-feature set used by all candidate models. LASSO, RFE, and permutation importance were complementary screens; the near-plateau in RFE performance supported an eight-feature model size. Binary variables were encoded 0/1 and surgical procedure was one-hot encoded during model fitting; individual dummy columns were not counted as separate candidate predictors. HVA, hallux valgus angle; IMA, intermetatarsal angle; DMAA, distal metatarsal articular angle; TMT, tarsometatarsal joint.

### 3.3 Performance comparison and selection of candidate models

In nested cross-validation of the development cohort conditional on the fixed eight-feature set, ROC-AUC was 0.908 for LR and 0.906 for SVM, while PR-AUC was 0.766 and 0.757, respectively. SVM had a lower Brier score (0.083 vs 0.132) and, at the locked classification threshold, higher specificity, F1 score, and balanced accuracy. Because discrimination was essentially comparable, these results do not establish statistical superiority of SVM over LR. SVM was retained for the locked temporal-validation analysis because its development-cohort probability error and overall performance profile were more favorable. Candidate-model confidence intervals and model-specific calibration intercept/slope estimates were not available in the retained reporting output, so the comparison is presented descriptively rather than as a formal superiority test (Table 3, Figure 4).

**Table 3:** Performance comparison of five machine learning models in the development cohort.

Model	ROC-AUC	PR-AUC	Brier	Sensitivity	Specificity	F1	Balanced accuracy
LR	0.908	0.766	0.132	0.819	0.798	0.605	0.809
RF	0.856	0.689	0.114	0.833	0.773	0.588	0.803
XGBoost	0.860	0.664	0.110	0.806	0.757	0.560	0.781
LightGBM	0.844	0.634	0.112	0.819	0.738	0.551	0.779
SVM	0.906	0.757	0.083	0.819	0.833	0.641	0.826

All models used the same fixed eight-variable feature set. Nested cross-validation (5 outer folds and 3 inner folds) was used for model tuning and evaluation within the development cohort; feature selection itself had been performed once in the development cohort before this model comparison.

**Figure 4:**
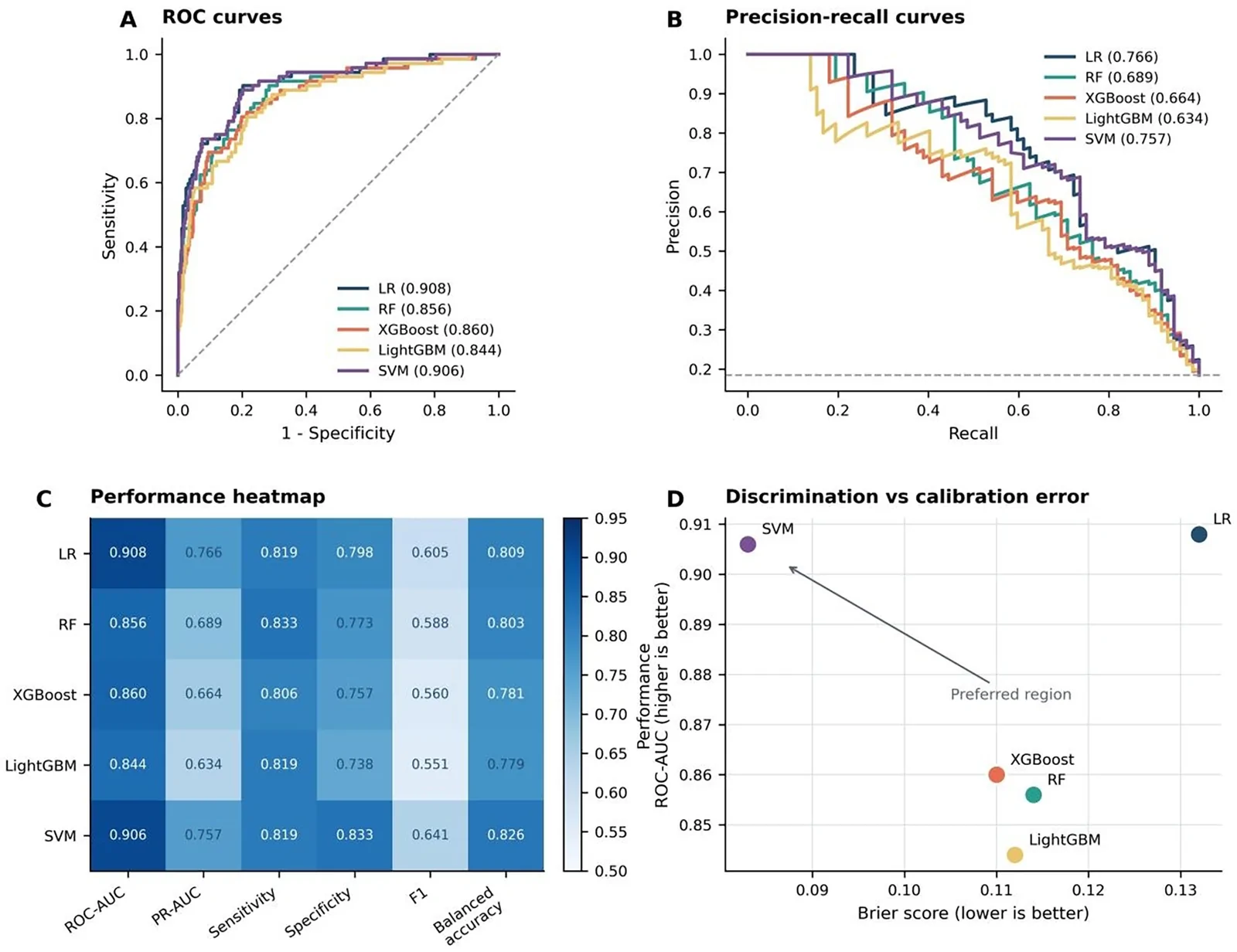
Performance comparison of five machine learning models under a unified 8-variable feature set; **(A)** ROC curves for each model; **(B)** precision–recall curves; **(C)** heatmap of key performance metrics—including ROC-AUC, PR-AUC,sensitivity,specificity,F1-score, and balanced accuracy; **(D)** joint comparison of ROC-AUC and brier score.

### 3.4 Temporal validation, calibration, and DCA

In the independent temporal-validation cohort (2024-2025), the locked SVM achieved an ROC-AUC of 0.944 (95% CI 0.908–0.973) and PR-AUC of 0.805 (95% CI 0.674–0.903); sensitivity was 0.892, specificity 0.842, PPV 0.550, NPV 0.973, F1 score 0.680, balanced accuracy 0.867, and Brier score 0.071 (95% CI 0.048–0.092) (Table 4). The development-cohort calibration intercept was approximately −0.093 and slope approximately 0.979; in the temporal-validation cohort, the intercept was 0.234 and slope 1.279. The positive validation intercept indicates a systematic calibration offset, while a slope >1 is consistent with some under-dispersion of predicted risks; therefore, the strong discrimination should not be interpreted as perfect calibration, and recalibration may be required in external populations (10, 18). The calibration plot in Figure 5B displays observed versus predicted recurrence across the plotted probability range. DCA showed positive net benefit relative to treat-all and treat-none across threshold probabilities of 0.05–0.50 (Figure 5), but this is interpreted as potential decision utility rather than evidence that model-guided management improves outcomes. The higher ROC-AUC in the later cohort than in development out-of-fold evaluation should be interpreted cautiously because the temporal-validation cohort contained only 37 recurrence events and the difference may reflect sampling variation or case-mix differences rather than improved generalizability.

**Table 4:** Independent temporal-validation performance of the final SVM model.

Metric	Estimate	95% CI
ROC-AUC	0.944	0.908–0.973
PR-AUC	0.805	0.674–0.903
Sensitivity	0.892	0.781–0.976
Specificity	0.842	0.787–0.891
PPV	0.550	0.413–0.676
NPV	0.973	0.947–0.994
F1 score	0.680	0.556–0.779
Balanced accuracy	0.867	0.806–0.919
Brier score	0.071	0.048–0.092
Calibration intercept	0.234	—
Calibration slope	1.279	—

After the development analysis was locked, the final SVM and its hyperparameters were applied once to the independent temporal-validation cohort (2024-2025, *n* = 208). The cohort was not used for feature screening, model training, hyperparameter tuning, or threshold selection. The classification threshold was fixed at 0.172 before temporal validation.

**Figure 5:**
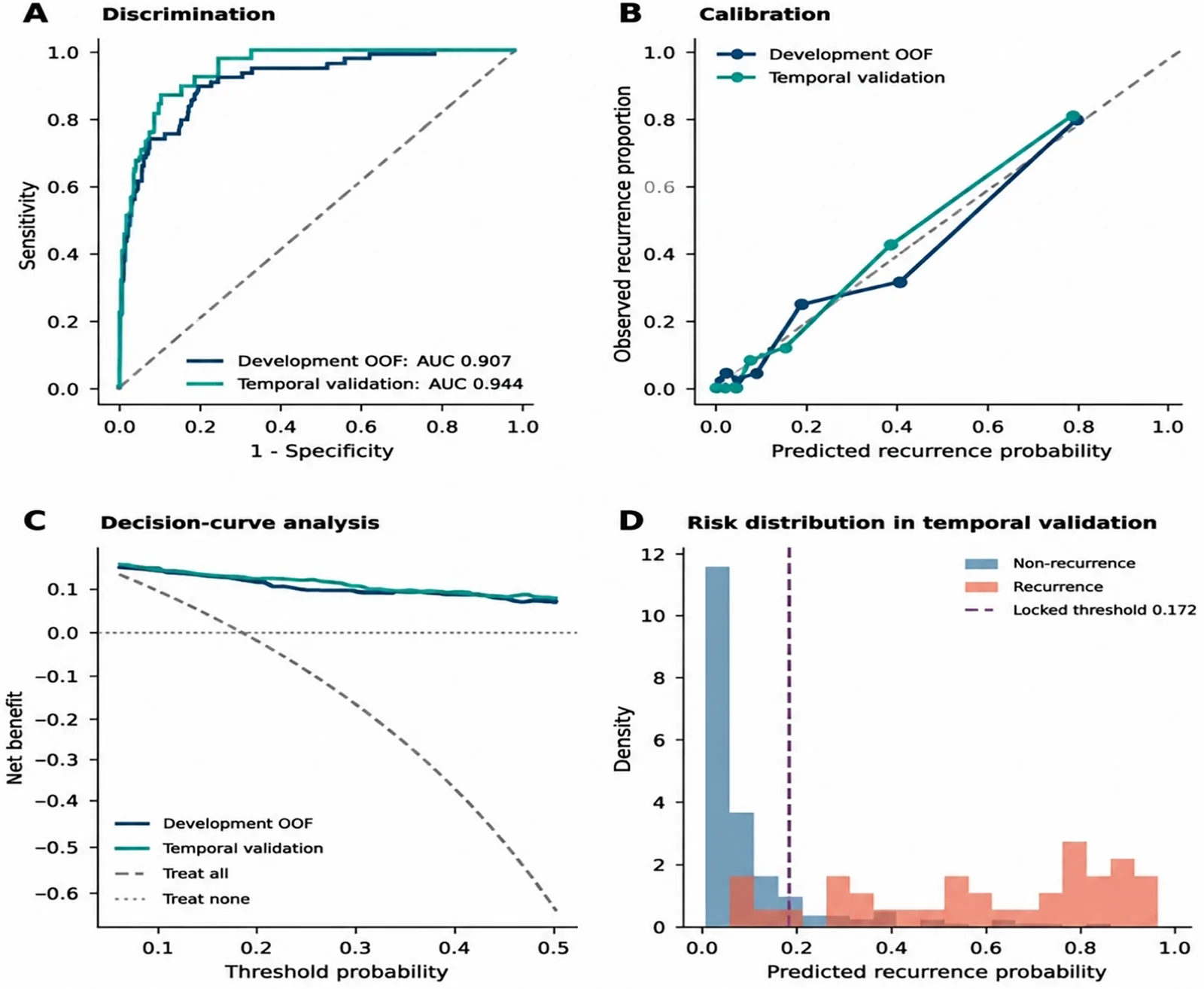
Temporal validation, calibration, and clinical utility of the final locked SVM model: **(A)** ROC curves for development out-of-fold predictions and the temporal-validation cohort; **(B)** calibration curves for both cohorts; **(C)** decision curve over threshold probabilities 0.05-0.50; **(D)** predictive-probability distribution in temporal validation, with the locked development-cohort threshold of 0.172 indicated by the vertical dashed line.

### 3.5 SHAP and individual explanation

SHAP global analysis showed the highest mean absolute SHAP value for 6-week postoperative IMA (0.0755), followed by 6-week HVA (0.0660), 6-week sesamoid grade (0.0647), preoperative HVA (0.0438), preoperative IMA (0.0362), 6-week DMAA (0.0343), preoperative DMAA (0.0273), and age (0.0168) (Figure 6). In representative high-risk recurrence case HV0430, the predicted probability was 93.5%; the 6-week IMA of 10.3°, HVA of 16.0°, and sesamoid grade 3 contributed to the model prediction toward higher recurrence risk. In low-risk non-recurrence case HV0552, the predicted risk was 0.1%; the 6-week IMA of 2.7° and sesamoid grade 1 contributed primarily toward lower predicted risk (Figure 7).

**Figure 6:**
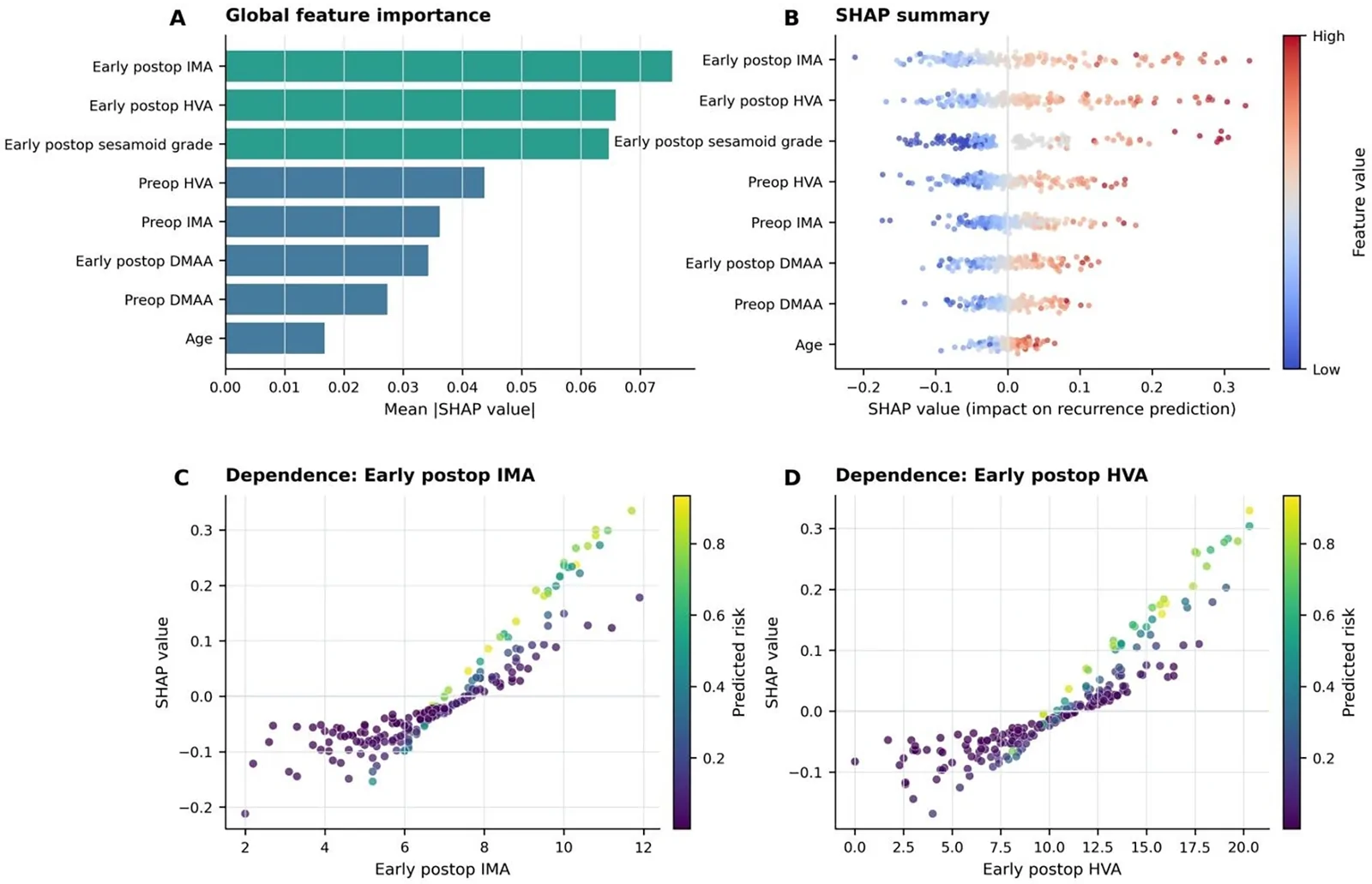
SHAP-based global interpretation of the SVM model in the validation cohort; **(A)** importance scores of the eight variables ranked by mean absolute SHAP value; **(B)** beeswarm plot (distribution of contribution magnitude, direction, and values);**(C)** IMA dependency plot at 6 weeks postoperatively; **(D)**HVA dependency plot at 6 weeks postoperatively.

**Figure 7:**
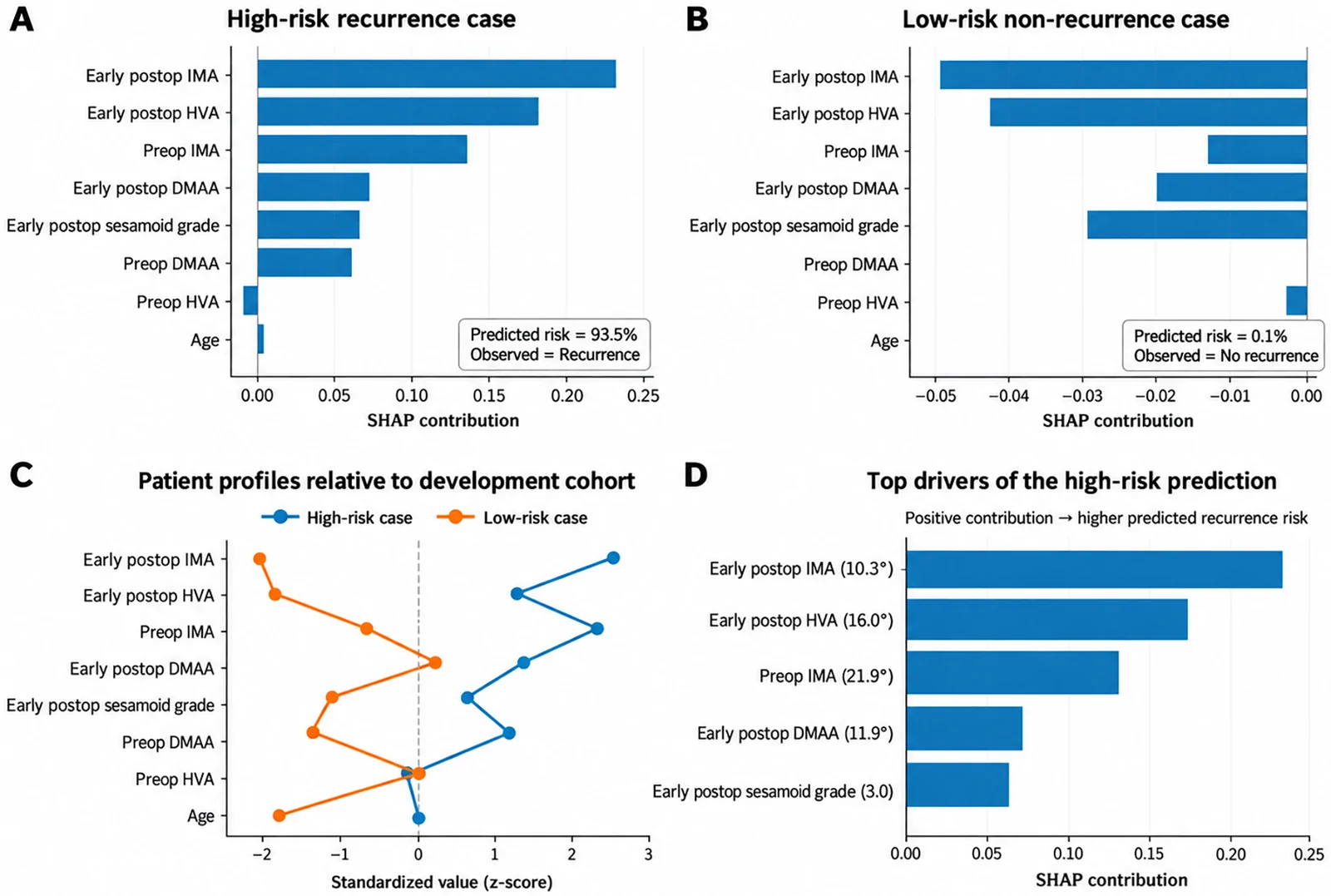
Individual-level model interpretation for representative high-risk and low-risk cases; **(A)** SHAP local contributions for representative high-risk recurrent patients; the driving effect of each variable on predicting the recurrence probability is displayed according to its contribution magnitude; **(B)** SHAP local contributions for representative low-risk non-recurrent patients; **(C)** Standardized feature profiles of the eight final predictive variables for high-risk and low-risk patients relative to their distribution in the development cohort; **(D)** The variable with the largest contribution to the prediction of high-risk patients and its corresponding original clinical measurement values.

## 4 Discussion

Hallux valgus is a common three-dimensional forefoot deformity characterized by medial deviation of the first metatarsal, lateral deviation of the hallux, and displacement of the sesamoid complex. Its prevalence is higher in women and older populations (2). For symptomatic HV that does not respond to conservative treatment, surgical correction remains an important treatment option, with techniques including Chevron, Scarf, proximal metatarsal osteotomy, and Lapidus procedures (22). Postoperative radiographic recurrence, frequently defined using an HVA threshold around 20°, remains an important long-term issue (5). Previous studies have identified multiple associated factors, including early postoperative HVA, IMA, and sesamoid position (23, 24). Because these factors may interact, machine-learning methods may be useful for integrating multidimensional predictors. Zhao et al. (8) developed an HV recurrence model using dual-center data, with an SVM external-validation AUC of 0.88. The present study used single-center real-world data, routine perioperative clinical and radiographic parameters, temporal validation, nested cross-validation for candidate-model comparison conditional on a locked feature set, and *post-hoc* SHAP-based interpretation. We hypothesized that a multiparameter model incorporating 6-week residual-deformity measures would predict fixed 1-year radiographic recurrence and that early postoperative IMA, HVA, and sesamoid grade would be major contributors to model predictions.

This study included 597 patients and evaluated a recurrence-prediction model with *post-hoc* SHAP-based interpretation after hallux valgus surgery. The main findings were that routine perioperative clinical and imaging parameters provided useful discrimination of fixed 1-year radiographic recurrence; SVM was retained from development-model comparison and achieved an ROC-AUC of 0.944 and PR-AUC of 0.805 in the independent temporal-validation cohort; and 6-week IMA, HVA, and sesamoid grade had the largest SHAP contributions. These SHAP results identify variables that strongly influence model predictions but do not establish that the variables are causal determinants of recurrence. The temporal-validation result should also be interpreted in the context of only 37 recurrence events. In the development cohort, only 72 recurrence events were available for a workflow involving feature screening, five candidate algorithms, and nonlinear model tuning. The effective complexity of an RBF-SVM cannot be summarized simply by the eight retained input variables; it also depends on the kernel representation and fitted support vectors. Accordingly, both internal and temporal-validation performance may be sensitive to sampling variation, and the present results should be regarded as preliminary rather than evidence of clinical readiness.

The 1-year radiographic recurrence rate was 18.3%, lower than the 24.9% reported by Ezzatvar et al. (5), although recurrence estimates depend strongly on definition and follow-up window. The HVA >20° threshold used in the present study is a common radiographic definition in the hallux valgus literature (5, 25). Six-week IMA, HVA, and sesamoid grade ranked as the leading SHAP features, which is consistent with prior reports associating incomplete sesamoid reduction and residual angular deformity with later recurrence (24, 26). In the present study, these variables should be interpreted as strong predictive signals within the fitted model rather than as proof of causal effects. Their prominence supports careful assessment of residual angular deformity and sesamoid position at the early postoperative visit, while causal and treatment-effect questions require separate study designs.

Preoperative HVA, IMA, and DMAA remained in the final model, indicating that baseline deformity severity retained predictive information in addition to 6-week postoperative measures. The exclusion of other variables from the final set does not imply absence of etiological importance; it indicates only that they were not retained in this predictive specification. Multiple surgical techniques were represented, and surgical procedure was not retained as a core predictor. This should not be interpreted as evidence that procedures are equivalent or that technique is unimportant. Comparative evidence has assessed outcomes across different first-metatarsal osteotomy sites (27). Previous Scarf osteotomy studies have reported associations between first metatarsophalangeal joint incongruity, postoperative noncompliance, and recurrence (28, 29). A separate meta-analysis has compared Scarf and Chevron osteotomies in terms of IMA correction (30). Postoperative radiographic variables may partly reflect or mediate the effect of procedure selection and correction achieved. With only 37 recurrence events in the temporal-validation cohort distributed across several operative categories, procedure-specific or severity-stratified calibration and discrimination estimates would be too imprecise for reliable interpretation; adequately powered subgroup validation is therefore needed.

Compared with the machine-learning study by Zhao et al. (8), the present study also identified strong predictive contributions from preoperative deformity, postoperative IMA, and sesamoid position and observed good SVM discrimination. Zhao et al. reported an external-validation AUC of 0.88 in 198 feet from two centers, whereas the present temporal-validation cohort yielded an AUC of 0.944. These values arise from different populations, sample sizes, recurrence definitions, predictor sets, prevalence, outcome timing, and validation designs and therefore should not be interpreted as evidence that one model is superior. The present analysis additionally reports PR-AUC, Brier score, calibration, DCA, and SHAP-based individual explanations, while multicenter external validation remains necessary.

Model selection was not based on ROC-AUC alone. LR and SVM had nearly identical development discrimination (ROC-AUC 0.908 vs 0.906; PR-AUC 0.766 vs 0.757), while SVM had a lower Brier score and more balanced threshold-dependent metrics at the locked operating point. Because candidate-specific confidence intervals and full model-specific calibration parameters were not available in the retained output, we do not claim formal statistical superiority of SVM over LR; SVM was retained as the final model for temporal validation on the basis of the overall development performance profile. In the temporal-validation cohort, the calibration intercept of 0.234 and slope of 1.279 indicate that calibration was not perfect despite the low Brier score, with systematic offset and some under-dispersion of predicted risks. External recalibration may therefore be necessary before use in a new population. SHAP was used to describe how individual variables contributed to model predictions. Larger 6-week IMA, HVA, and residual sesamoid displacement contributed toward higher predicted recurrence risk in representative cases, but SHAP values should not be interpreted as causal effects. LIME-style local surrogate analysis was used only as a supplementary model-agnostic explanation and was not interpreted etiologically.

This study has several limitations. (1) The single-center retrospective design limits generalizability, and temporal validation cannot substitute for multicenter external validation. (2) The development cohort contained only 72 recurrence events and the temporal-validation cohort only 37, limiting precision and increasing the possibility of performance instability; the later cohort is particularly limited for calibration and procedure- or severity-specific subgroup assessment. The higher ROC-AUC observed in the later cohort may reflect sampling variation or case-mix differences and should not be interpreted by itself as proof of robustness. (3) The primary outcome was fixed at 12 months; therefore, the model does not address recurrence occurring after the 1-year window despite longer descriptive follow-up in some patients. The exact scheduling tolerance around the nominal 12-month visit was not preserved in the analytic archive. (4) The outcome was radiographic and did not incorporate patient-reported pain, function, or revision surgery. (5) The retained radiographic-reading record does not preserve observer identities/experience, blinding, duplicated readings, or a study-specific reliability sample; therefore, shared observer-related measurement error between radiographic predictors and outcome cannot be excluded (13, 14). (6) The unified eight-feature set was selected once using the full development cohort before nested candidate-model comparison. A fully nested workflow would repeat data-dependent feature selection within each outer training fold (31); our internal estimates are therefore conditional on the locked development-derived feature set and may retain some optimism. (7) The retained study archive does not include the complete hyperparameter search grids, random-seed log, per-outer-fold performance distributions, exact SHAP explainer configuration, exact probability-calibration implementation, rule used to derive the 0.172 threshold, criterion-specific screening/exclusion counts, bilateral-procedure handling details, standardized-difference summaries between temporal cohorts, or the scaling parameters required for a fully deployable model object. These items are acknowledged as reporting/reproducibility limitations rather than being assigned retrospective values. Multicenter validation with prospectively documented patient flow, observer reliability, subgroup calibration, fully nested feature selection, and a reproducible model release is required before clinical implementation.

## 5 Conclusion

This study developed an SVM model with *post-hoc* SHAP-based interpretation using eight routine clinical/perioperative imaging parameters for prediction of fixed 1-year radiographic recurrence after hallux valgus surgery. The model showed promising discrimination and probability performance in an independent temporal-validation cohort, while the limited number of recurrence events and reporting/reproducibility constraints warrant cautious interpretation. SHAP identified 6-week IMA, HVA, and sesamoid grade as leading contributors to model predictions. The model may support identification of patients who merit enhanced follow-up, but multicenter external validation, observer-reliability assessment, reproducible model release, and prospective impact evaluation are needed before clinical implementation.

## Data Availability

The original contributions presented in the study are included in the article/Supplementary Material, further inquiries can be directed to the corresponding author.
